# Ultrasound can increase biofilm formation by *Lactiplantibacillus plantarum* and *Bifidobacterium* spp.

**DOI:** 10.3389/fmicb.2023.1094671

**Published:** 2023-03-06

**Authors:** Angela Racioppo, Barbara Speranza, Clelia Altieri, Milena Sinigaglia, Maria Rosaria Corbo, Antonio Bevilacqua

**Affiliations:** Department of Agriculture, Food, Natural Resources and Engineering, University of Foggia, Foggia, Italy

**Keywords:** ultrasound, functional microorganisms, biofilm formation, adhesion, stability

## Abstract

The main goal of this research was to study the effect of an Ultrasound (US) treatment on biofilm formation of *Lactiplantibacillus plantarum* (strains c19 and DSM 1055), *Bifidobacterium animalis* subsp. *lactis* DSM 10140, *Bifidobacterium longum* subsp. *longum* DSM 20219, and *Bifidobacterium longum* subsp. *infantis* DSM 20088. From a methodological point of view, each microorganism was treated through six US treatments, different for the power (10, 30, or 50% of the net power, 130 W), the duration (2, 6, or 10 min) and the application of pulses (0 or 10 s). After the treatment, a biofilm of the strains was let to form on glass slides and the concentration of sessile cells was analyzed for 16 days. Biofilms formed by untreated microorganisms were used as controls. As a first result, it was found that US significantly increased the concentration of sessile cells of *B. longum* subsp. *infantis*, while for some other strains US treatment could not affect the formation of biofilm while improving its stability, as found for *L. plantarum* DSM1055 after 16 days. The variable mainly involved in this positive effect of US was the duration of the treatment, as biofilm formation and stability were improved only for 2 min-treatments; on the other hand, the effect of power and pulses were strain-dependent. In conclusion, the results suggest practical implication of a US pre-treatment for various fields (improvement of adhesion of microorganisms useful in food or in the gut, biomedical and environmental industries), although further investigations are required to elucidate the mode of action.

## 1. Introduction

Ultrasounds (US) are elastic waves that move through a medium producing compression (high pressure) or rarefaction (low pressure) and whose frequency is on average higher than that audible to the human ear (20 kHz) (Ojha et al., 2017). Depending on their use, US can be classified into high-power US (low frequency) and low-power US (high frequency) (Estivi et al., 2022). Low-power US are an excellent alternative to heat-based treatments because they avoid the negative effects of traditional processing (sensory and organoleptic decay), assuring at the same time the inactivation of spoiling and pathogenic microorganisms (Moosavi et al., 2021). In food, US have been used for a wide range of products and with very interesting purposes, including inactivation of microorganisms and enzymes, production of emulsions and nano-emulsions, extraction of bioactive compounds and oils from plant cells, and removal of microorganisms from surfaces (Bevilacqua et al., 2019a; Soltani-Firouz et al., 2019).

Among others, food matrix properties (intended as kind of material, and viscosity), temperature, intensity, power, and treatment time are parameters that influence the effect of US on processed products (Chavan et al., 2022). Depending on the intensity and duration of treatment, US could exert a dual effect on the microbial cell. High-intensity US could damage membranes causing viability loss and release of cellular components, while low-intensity US could stimulate bacterial metabolism (Bevilacqua et al., 2019a). First, this dual effect was reviewed by Erriu et al. (2014) for microbial biofilms, while recent studies have shown the positive effect of US on biofilm stability of *Acidipropionibacterium jensenii*, *Propionibacterium freudenreichi* (Bevilacqua et al., 2019b), *Limosilactobacillus reuteri* (Racioppo et al., 2017), and *Lacticaseibacillus casei* (Giordano and Mauriello, 2023).

However, there are a few data and many times the effects on the same species are controversial. For example, the penetration effect of low-intensity/low-frequency US in *Staphylococcus aureus* biofilms was studied by Wang et al. (2020), thus they found that this type of US significantly increased biofilm permeability, depending on time and ultrasound intensity. On the other hand, Yu et al. (2021) evaluated the effectiveness of high-frequency US against *S. aureus* biofilm; the results clearly indicated that at high intensity and frequency US could disrupt *S. aureus* clusters and cause cell lysis.

Historically, biofilm have been considered with a strong negative impact for food quality and health, as spoilers or pathogens can grow in sessile form (Guéneau et al., 2022); however, recently, their impact and significance has been re-evaluated, because biofilm produced by positive microorganisms could contribute to safety by counteracting pathogen growth through a variety of effects (competition for nutrients, production of antimicrobial compounds, anti-adhesive effects, microbial interference, etc.) (Guéneau et al., 2022).

Indeed, the ability of probiotics to colonize biotic and abiotic surfaces by forming biofilms could have great potential for human health and food safety. In the biomedical field, for example, a biofilm formed by probiotic microorganisms could be useful in hindering the development of infection-causing microorganisms, while in the food industry biofilms can be used to ensure the health safety of food products and the extension of their shelf life. In a recent study, Speranza et al. (2020) focused on *in vivo* metabolism of *Bifidobacterium longum* subsp. *infantis* and *L. reuteri*, and showed that these probiotics form biofilms able to control the growth of harmful bacteria.

The question beyond this research is if biofilm formation by positive bacteria could be modulated and increased by physical approaches; therefore, this research examines biofilm formation by *Lactiplantibacillus plantarum* and *Bifidobacterium* spp. and the possibility of a modulation of adhesion properties through an US treatment, also assessing the effect of the main treatment variables (power, duration, pulse) on biofilm formation and stability.

## 2. Materials and methods

### 2.1. Microorganisms

This study focused on two strains of *L. plantarum*, labeled as c19 (a wild strain isolated from Bella di Cerignola table olives and with some functional properties; Bevilacqua et al., 2010) and DSM 1055 (from the German Collection of Microorganisms, Braunschweig, Germany), and on the collection isolates *B. animalis* subsp. *lactis* DSM 10140, *B. longum* subsp. *longum* DSM 20219, and *B. longum* subsp. *infantis* DSM 20088.

The strains of *L. plantarum* were stored at −20°C in MRS broth (Oxoid, Milan, Italy) added with 33% of sterile glycerol (J.T. Baker, Milan, Italy), while *Bifidobacterium* spp. were stored at −20°C in MRS broth supplemented with 0.5% cysteine (cMRS) (Sigma-Aldrich, Milan, Italy) added with 33% of sterile glycerol; cysteine creates a reducing environment for *Bifidobacteria*. Before each assay, the microorganisms were grown under anaerobic conditions either in MRS broth or cMRS broth, incubated at 37°C for 24 h in jars. Then, the cultures were centrifuged at 4,000 × *g* for 10 min at 4°C; the supernatant was discarded, and the pellet suspended in distilled water.

### 2.2. US—Treatment

Bacteria suspension at ∼10^7^ CFU/ml, prepared as reported in section “Microorganisms,” were treated through a VC Vibra Cell Ultrasound equipment, model VC 130 (Sonics and Materials Inc., Newtown, CT, USA; net power, 130W) as follows. 20 ml of bacterial suspension were put in 50 ml sterile tubes; then, ultrasonic probe was placed 1–2 cm below the surface and in the middle of tube. Each strain was US-treated through 6 different combinations, different for power (from 10 to 50% of the net power of equipment, i.e., 130 W), pulse (from 0 to 10 s), and duration of the treatment (from 2 to 10 min). These three variables were combined through a centroid approach, as reported in Table 1. According to the centroid approach each variable was set to 3 different levels, identified by the codes 0 (minimum), 1 (maximum), 0.5 (mean value). Table 1 shows the 6 combinations of the centroid and the control (i.e., an additional combination in which power, treatment time, and pulse were set to 0, that is microorganism not treated through US). US was done in aerobic conditions.

**TABLE 1:** US treatments tested on *L. plantarum* and *Bifidobacterium* spp.

	Coded values			Real values		
	**Power**	**Time**	**Pulse**	**Power (%)**	**Time (min)**	**Pulse (s)**
1	1	0	0	50	2	0
2	0	1	0	10	10	0
3	0	0	1	10	2	10
4	0.5	0.5	0	30	6	0
5	0.5	0	0.5	30	2	5
6	0	0.5	0.5	10	6	5
Control	–	–	–	–	–	–

Before each treatment, the ultrasonic probe was washed with sterile distilled water, ethanol at 70% and again with distilled water; immediately after processing, the sample was cooled in ice. After each US treatment, a plate count was performed on MRS agar (*Lactobacilli*) or cMRS agar (*Bifidobacteria*), incubated at 37°C for 48 h; plates were incubated under anaerobic conditions (jars and anaerobic kit).

### 2.3. Biofilm formation

Bacterial suspensions treated through US and control strains were used to inoculate broth containing glass slides (25.4 mm × 76.2 mm), as surface adhesion for biofilm formation. Before each experiment, it is important to perform a preliminary treatment on glass slides to remove any fingerprints, grease and other impurities that might be present on the material. Thus, the slides were washed with acetone for at least 30 min, rinsed in distilled water and immersed in 1 N NaOH for 1 h. After a final rinse in distilled water, the slides were allowed to air dry. Finally, the slides were autoclaved at 121°C for 15 min before use. After the treatment, the slides were placed in 50 ml-tubes (one slide per tube) containing 45 ml of MRS broth (*Lactobacilli*) or cMRS broth (*Bifidobacteria*). The final step for sample preparation is the inoculation of tubes with slides and broth with US bacteria, adding 200 μl of the suspensions prepared as reported in section “US—Treatment” (US-treated suspensions and untreated bacteria) to gain an initial inoculum of ∼10^5^ CFU/ml).

After preparation and inoculation, samples were incubated at 37°C in jars; for each microorganism 7 different combinations were tested (6 US combinations and untreated microorganism).

After 1, 5, 7, 9, 12, and 16 days, slides were aseptically removed from the culture medium, rinsed with sterile distilled water to remove unattached cells, and placed in a tube containing 40 ml of sterile saline solution (0.9% NaCl). Then, the samples were sonicated at 20% power for 3 min to promote detachment of cells from the surface (Speranza et al., 2009). Microbiological analyses were done on this saline solution on either MRS agar or cMRS agar, incubated at 37°C for 48 h in jars.

### 2.4. Statistical analysis

All tests were performed in duplicate on two independent samples for each combination and for each strain. Cell concentration recovered from saline solution (section “Biofilm formation”) was converted to log CFU/cm^2^ through the following formula:


C1=C2⁢V*⁢SS⁢A

where C_1_ is the viable count expressed in log CFU/cm^2^; C_2_ is the viable count expressed as log CFU/ml; VS is the volume during the sonication treatment (40 ml) and SA the area of adhesion tested (39 cm^2^, surface area of the slide considering both sides).

Data of *L. plantarum* were analyzed through a multifactorial Analysis of Variance, using strain (c19 or DSM 1055), age of biofilm or sampling time (1, 5, 7, 12, and 16 days) and combinations of US treatment (control, or combinations from 1 to 6) as categorical predictors; Fisher LSD test was used as the *post-hoc* test. Statistic was done through the software Statistica for Windows (Statsoft, Tulsa, OK, United States).

Afterwards, data at day 1 for *B. infantis* and after 16 days for *L. plantarum* DSM 1055 were analyzed through a multiple regression approach through the option DoE/mixture design of the software Statistica to assess the significance of the three parameters of US treatment (power, pulse, and duration).

## 3. Results

### 3.1. Effect of US treatment on biofilm of *Bifidobacterium* spp.

Table 2 shows the viable count of US-treated bacteria (log CFU/ml), compared to the control (CNT, untreated strains), immediately after the treatment; US did not reduce cell count, which was between 7.65 ± 0.068 log CFU/ml (*L. plantarum*) and 6.86 ± 0.02 log CFU/ml (*Bifidobacterium* spp.) in the control, against values of 7.71 ± 0.03 log CFU/ml (*L. plantarum*) and 6.23 ± 0.15 log CFU/ml (*Bifidobacterium* spp.) recovered after the most drastic treatment (combination 1, 50% of the net power, 2 min, pulses at 0 s), thus suggesting that US did not affect the viability of the test strains and could be used for a positive modulation of biofilm formation.

**TABLE 2:** Viable count (log CFU/ml) of US-treated bacteria compared to the control (untreated microorganism, CNT) immediately after the treatment.

	Combination						
**Strains**	**CNT**	**1**	**2**	**3**	**4**	**5**	**6**
*L. plantarum* c19	7.59 ± 0.02	7.55 ± 0.0	7.66 ± 0.05	7.54 ± 0.07	7.48 ± 0.01	7.59 ± 0.00	7.52 ± 0.03
*L. plantarum* DSM 1055	7.65 ± 0.07	7.72 ± 0.03	7.69 ± 0.21	7.82 ± 0.12	7.58 ± 0.03	7.53 ± 0.05	7.88 ± 0.0
*B. longum* subsp. *infantis*	6.73 ± 0.11	6.63 ± 0.21	6.89 ± 0.40	7.10 ± 0.08	6.81 ± 0.47	7.28 ± 0.04	7.07 ± 0.13
*B. longum* susp. *longum*	6.47 ± 0.66	6.54 ± 0.60	6.07 ± 0.01	6.12 ± 0.11	6.17 ± 0.06	6.14 ± 0.01	6.97 ± 0.27
*B. animalis* subsp. *lactis*	6.86 ± 0.06	6.22 ± 0.15	7.24 ± 0.89	7.20 ± 0.26	7.23 ± 0.09	7.46 ± 0.03	7.19 ± 0.02

For each row, the differences were not significant (one-way ANOVA and Fisher LSD test, *P* > 0.05). Mean values ± standard deviation.

Generally, *Bifidobacteria* and *L. plantarum* showed different adhesion properties, with a very low stability of biofilm for *Bifidobacterium* spp.; therefore, the strains were separately analyzed to avoid a confounding effect due to the higher count of sessile cells in *L. plantarum*.

Ultrasound treatment did not affect the adhesion properties of *B. longum* subsp. *longum* and *B. animalis* subsp. *lactis* strains. After 24 h, the concentration of sessile cells was 6.3 and 5.85 log CFU/cm^2^ for *B. longum* subsp. *longum* and *B. animalis*, respectively, and US treatment did not change this trend; in addition, the count of sessile cells experienced a strong reduction in all samples and was below the detection limit after 5–7 days (data not shown).

On the other hand, a significant effect was found for *B. longum* subsp. *infantis* (Table 3); after 1 day, biofilm produced by US-treated microorganism was at higher levels than the control (5.0 log CFU/cm^2^ vs. 5.80–6.87 log CFU/cm^2^) and the highest count was found in the combination 1 (treatment at 50% of power, for 2 min, no pulse). Moreover, this combination also resulted in a higher stability of biofilm, with a residual concentration of sessile cells at 3.70 log CFU/cm^2^ after 9 days, while for untreated microorganism or for cells treated with other US combinations sessile cells were below the detection limit.

**TABLE 3:** Concentration of sessile cells (log CFU/cm^2^) of *B. longum* subsp. *infantis*, preliminary treated through US.

	Biofilm age			
Biofilm produced from US-treated strain	1 day	5 days	7 days	9 days
CNT (untreated microorganism)	5.01 ± 0.23a	4.36 ± 0.67	3.79 ± 0.0	ND**
1*	6.87 ± 1.03b	4.19 ± 0.28	3.69 ± 0.45	3.71 ± 0.0
2	6.24 ± 0.0a,b	ND	ND	ND
3	6.13 ± 0.27ab	ND	ND	ND
4	6.47 ± 0.0a,b	ND	ND	ND
5	5.80 ± 0.33a	ND	ND	ND
6	6.08 ± 0.01a	3.82 ± 0.0	ND	ND

CNT, control. Mean values ± standard deviation; in the column for the data at day 1, letters indicate significant differences (one-way ANOVA and Fisher LSD test, *P* < 0.05).

*Combination of the centroid; **not detected.

### 3.2. Effect on US treatment on biofilm of *Lactiplantibacillus plantarum*

As reported elsewhere *L. plantarum* strains showed different trends; moreover, the stability of biofilm was higher than that found for *Bifidobacteria*. Finally, the two strains behaved in a different way, thus requiring a preliminary standardization of data as biofilm detachment or reduction of biofilm count compared to the day 1; therefore, data for these two strains should be read as reduction of the count of sessile cells throughout time.

Modeling was done through MANOVA; the table of standardized effects points out that all predictors, as individual or interactive terms (combination, strain, age of biofilm, combination × strain, combination × age of biofilm, strain × age of biofilm, combination × strain × age of biofilm), were found to be significant; however, their statistical weights were different. The most important factor, as an individual term, was the age of biofilm, followed by the strain, and finally by the combination of US-treatment; on the other hand, the most important interactive term was “strain × age of biofilm” (Table 4).

**TABLE 4:** Standardized statistical effects related to individual and interactive terms of the strain, US treatment, and biofilm age of *L. plantarum* c19 and DSM 1055.

	Effects
Combination	3.09
Strain	130.19
Biofilm age	597.72
Combination × strain	5.61
Combination × age	3.40
Strain × age	99.90
Combination × strain × age	4.71

The results were obtained through a multifactorial ANOVA (analysis of variance).

The estimation of the quantitative effects of predictors is possible through the decomposition of the statistical hypothesis, which does not show real trends but the mathematical correlation of each predictor vs. the dependent variable.

**FIGURE 1:**
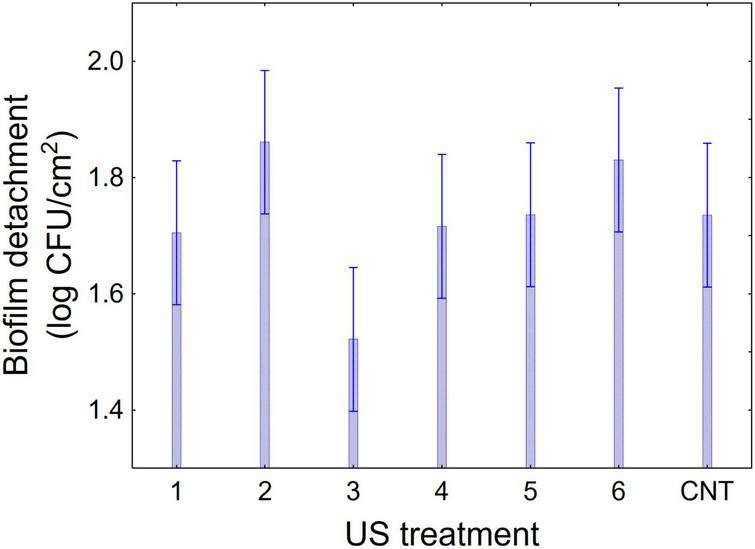
Decomposition of statistical hypothesis related to the individual term of US-treatment on the detachment of biofilm (log CFU/cm^2^) of *L. plantarum* c19 and DSM 1055. Mean values ± 95% confidence interval.

**FIGURE 2:**
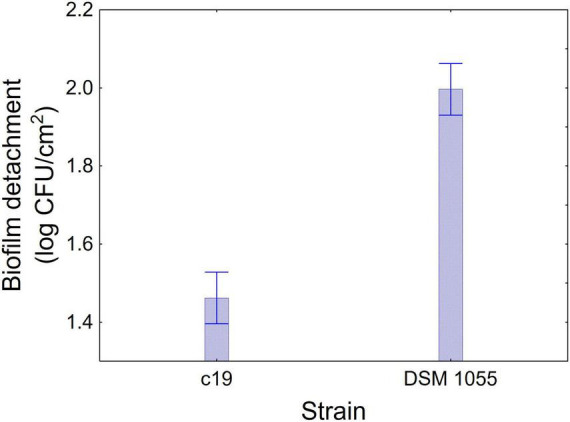
Decomposition of statistical hypothesis related to the individual term of strain on the detachment of biofilm (log CFU/cm^2^) of *L. plantarum* c19 and DSM 1055. Mean values ± 95% confidence interval.

**FIGURE 3:**
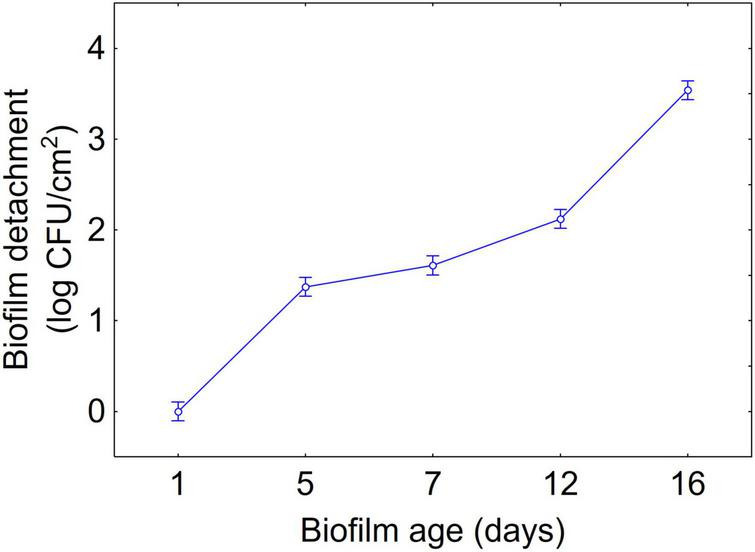
Decomposition of statistical hypothesis related to the individual term of biofilm age on the detachment of biofilm (log CFU/cm^2^) of *L. plantarum* c19 and DSM 1055. Mean values ± 95% confidence interval.

Figures 1–3 show the decomposition of the statistical hypothesis for the individual effects of predictors (combination of US treatment, strain, and age of biofilm). Regarding the effect of the combination (Figure 1), the reduction of sessile cells (biofilm detachment) was minimum in combination 3 (power, 10%; duration of the treatment, 2 min; pulse, 10 s), while the other combinations did not show significant differences compared to control. The two strains produced a biofilm with a different stability, as generally *L. plantarum* DSM 1055 experienced a higher biofilm detachment (ca. 2.0 log CFU/cm^2^) (Figure 2); as expected, biofilm age negatively affected its stability, as the detachment increased over time (Figure 3).

**FIGURE 4:**
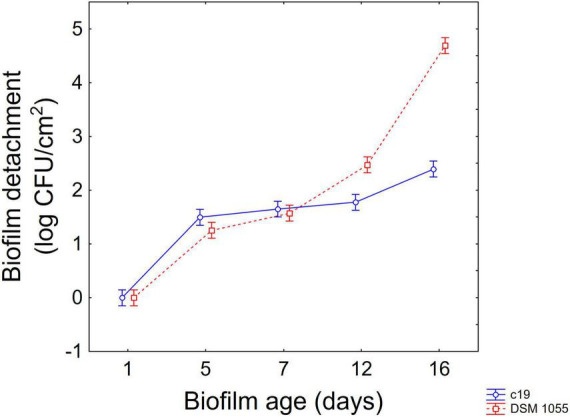
Decomposition of statistical hypothesis related to the interactive term “strain × biofilm age” on the detachment of biofilm (log CFU/cm^2^) of *L. plantarum* c19 and DSM 1055. Mean values ± 95% confidence interval.

**FIGURE 5:**
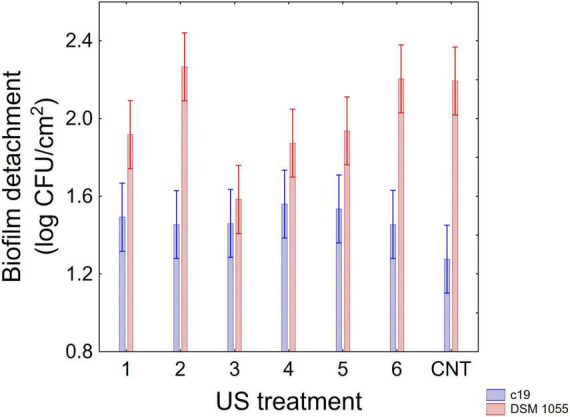
Decomposition of statistical hypothesis related to the interactive term “strain × US treatment” on the detachment of biofilm (log CFU/cm^2^) of *L. plantarum* c19 and DSM 1055. Mean values ± 95% confidence interval.

The approach of the decomposition of the statistical hypothesis was also used for the estimation of interactive terms. Figures 4, 5 show two-way interactions, namely for the interactions strain × age of biofilm (Figure 4) and combination of US treatment × strain (Figure 5).

The first interactive term highlights the higher stability of biofilm produced by the strain c19 mainly after 12 and 16 days, while for the different combinations the decomposition of the statistical hypothesis points out a higher stability of c19 biofilm in the control and in the combination 2 and 6.

**FIGURE 6:**
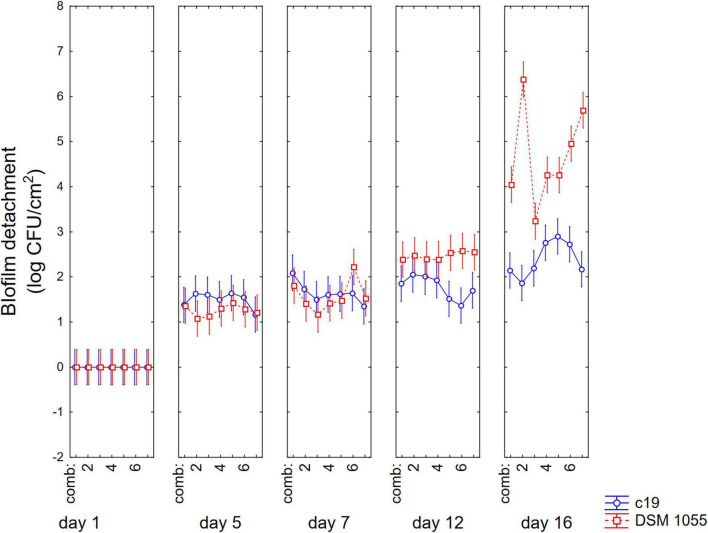
Decomposition of statistical hypothesis related to the interactive term “strain × biofilm age × US treatment” on the detachment of biofilm (log CFU/cm^2^) of *L. plantarum* c19 and DSM 1055. Mean values ± 95% confidence interval.

Finally, Figure 6 shows 3-way interaction, that is the actual values; after 12 days significant differences were observed between the two strains in all combinations, showing a higher detachment of the biofilm for the strain DSM 1055. However, for this strain, a US treatment before biofilm formation is crucial, as biofilm detachment after 16 days was 5.5. log CFU/cm^2^ in the control, 3.2 log CFU/cm^2^ for the microorganism treated with the combination 3 and 4.4 log CFU/cm^2^ when the microorganism had been treated with combinations 1, 4, and 5.

### 3.3. Significance of power, duration of US treatment and pulse on biofilm stability

Due to the significant effect of US on the viable count of sessile cells at day 1 for *B. longum* subsp. *infantis* and at day 16 for *L. plantarum* DSM 1055, the data for these sampling points were standardized as difference of sessile cells between US-treated microorganisms and untreated microorganisms and analyzed through a multiple regression procedure to understand how the three parameters of US (power, duration of the treatment, and pulse) could improve biofilm stability.

The first result of this approach is a set of standardized effects (Fisher-test values), which allows us to understand which variables were significant and which, among them, exerted the strongest effect. From a statistical point of view, for *B. longum* subsp. *infantis* the most important term was the power (*F*-test, 5.76), followed by the duration of the treatment (*F*-test, 3.81), and pulse (*F*-test, 3.47); the interactive terms (power × time, power × pulse, pulse × time) were not significant. In the case of *L. plantarum*, the most significant terms were power (*F*-test, 2.99) and pulse (*F*-test, 2.61).

**FIGURE 7:**
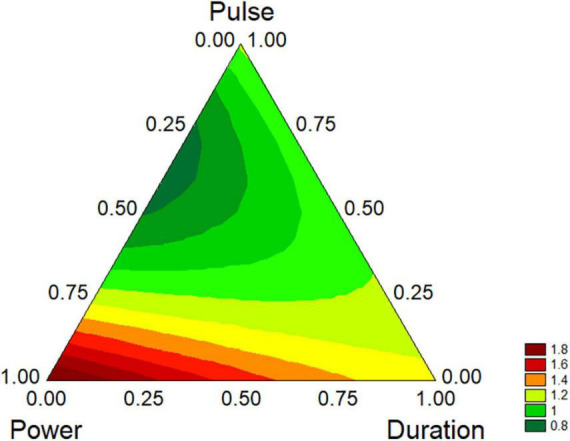
Triangular plots for the effect the factors of US-treatment (power, duration, and pulse) on biofilm stability compared to control (untreated microorganism) for *B. longum* subsp. *infantis* at day 1. The variables are reported as coded values (see Table 1).

**FIGURE 8:**
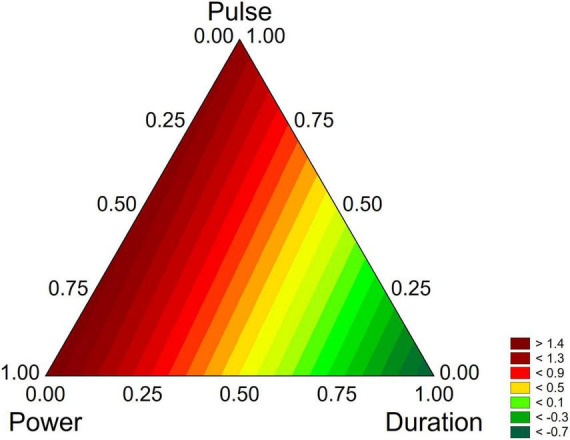
Triangular plots for the effect the factors of US-treatment (power, duration, and pulse) on biofilm stability compared to control (untreated microorganism) for *L. plantarum* DSM 1055 at day 16. The variables are reported as coded values (see Table 1).

However, the standardized effects show only the significance of the variables and do not allow the estimation of their quantitative effect. A better description can be obtained through the triangular plots; for *B. longum* subsp. *infantis* the triangular plot points out a higher value of biofilm for power-coded values around 1 (50% of net power), time 0 (treatment for 2 min), pulse 0 (0 s) (Figure 7). For *L. plantarum* DSM 1055 the highest difference US-treated strain vs. control (that is a higher stability of biofilm after US treatment) was found for the highest values of power or pulse (50% power and pulse at 10 s) or with the combination 0.5 power+0.5 pulse, as coded values, corresponding to real values of 30% of net power and 6 s of pulse, while an increase of the duration of the treatment exerted a detrimental effect on biofilm stability (Figure 8).

## 4. Discussion

Bacterial adhesion could be strongly influenced by physical treatments such as US, as reviewed by Erriu et al. (2014) and Bevilacqua et al. (2019a). The first aspect to consider when applying US to probiotic or useful microorganisms is the effect on viability and the results of the first step showed that the treatments tested never reduced the viability of strains, even after the most drastic combination.

As reported elsewhere, US could produce different effects, mainly depending on the energy loaded in the system and the energy of US is a function of several parameters, including intensity, frequency, and pulses (Jomdecha and Prateepasen, 2010). US generally act through sonoporation and cavitation (Ojha et al., 2017); it is reliable to assume that low intensity US only produce sub-lethal injuries, which could promote the release of cellular components, and an increased rate of exchanges with environment, thus promoting microbial growth (Avhad and Rathod, 2015; Dai et al., 2017; Bevilacqua et al., 2019a); conversely, high-intensity treatments cause the formation of pores and cavities, resulting in an uncontrolled release of cell components and thus cell death (Ojha et al., 2017).

Erriu et al. (2014) reviewed the impact of US on bacterial biofilm, and they reported a positive modulation of this trait; however, there are a few data on this topic and the mechanisms beyond this phenomenon are still unknown.

The first evidence stressed by the results of this paper is the strong strain-dependence of the effect, as it was found only on *B. longum* subsp. *infantis* and on *L. plantarum* DSM 1055; moreover, strain dependence is also reflected in the combinations acting on the two microorganisms (50% power/2 min/no pulse for *B. longum*; 50% power/pulse at 10 s or 30% power/pulse at 6 s for *L. plantarum* DSM 1055).

Strain dependence is probably related to the effect of US on the outer layers of cells, as it is known that the first targets are capsule and cell wall (Gao et al., 2014a,b; Krasovitski et al., 2011); therefore, differences in the composition of these layers could result in a different effect of US or the need of different combinations to exert similar actions.

Apart from the positive effect, the different results on *Bifidobacteria* and *L. plantarum* also suggest that US could positively act on two different properties of biofilm: formation and stability.

Biofilm formation is the result of a complex phenomenon, also involving the interaction between bacterial and adhesion surfaces; in this mechanism, hydrophobicity plays a fundamental role as an unspecific adhesion to hydrophobic surfaces is the first phase for many processes of biofilm formation (de Wouters et al., 2015). This effect on hydrophobicity could be the reason beyond the action on *Bifidobacteria*, as in the past an increased cell surface hydrohobicity of *Limosilactibacillus reuteri* and *Propionibacteria* was found by authors after US treatments (Racioppo et al., 2017; Bevilacqua et al., 2019b). This effect on hydrophobicity was also reported for *Lacticaseibacillus casei* ATCC 393 (Giordano and Mauriello, 2023), also coupled with an increased membrane permeability.

In addition to the increased hydrophobicity, the positive effect on biofilm formation could be explained by an aggregating effect (increased aggregation) found in *Lactobacillus acidophilus*, *Lacticaseibacillus casei*, and *Lactococcus lactis* subsp. *cremoris* from Tabatabaie and Mortazavi (2008) after treatment with US. Autoaggregation is a desirable property since it results in increased adhesion of microbial cells to the intestinal mucosa, providing advantages in colonization of the gastrointestinal tract (García-Cayuela et al., 2014; Trunk et al., 2018), as well as in the ability to survive in harsh environments. However, this effect on auto-aggregation is controversial and conflicting results have been found in the literature, as stressed by Giordano and Mauriello (2023).

Finally, US is known to increase cell permeability, which results in the passive diffusion of quorum-sensing protein signaling molecules that can stimulate biofilm formation (Kamaraju et al., 2011; Fitzgerald et al., 2018).

On the other hand, the positive effect on biofilm stability, mainly found on *L. plantarum* DSM 1055, could also involve other phenomena, like the increased nutrients transport to deeper layers of the biofilm (Peterson and Pitt, 2000; Erriu et al., 2014), as well as by a probable effect on membrane permeability, and exopolysaccharide production (Khadem et al., 2020). In fact, it is reliable to assume that a good diffusion of nutrients across biofilm could delay cell aging, and consequently their detachment from the adhesion surface. Erriu et al. (2014) also postulated that ultrasound could increase the oxygen rate in the deeper layer of biofilm; this effect on oxygen rate could be related to the increased biofilm stability, at least in *L. plantarum*. In this species, in fact, it is possible the shift to an aerobic metabolism (Zotta et al., 2017), which could improve stress resistance (Guidone et al., 2013); another effect could be the improvement of viability after a long-term starvation (Zotta et al., 2012, 2013). Finally, a postulated effect of oxygen, indirectly linked to adhesion at least on a recombinant strain of *Lacticaseibacillus casei*, was an increased yield on EPS production (Li et al., 2015).

In conclusion, a low intensity US treatment could improve adhesion properties of *Lactobacilli* and *Bifidobacteria*, depending on the power of the treatment, its duration, and the use of pulse.

Concerning the duration, a short exposure (2 min) could positively affect biofilm formation, while the results on pulses were controversial, as biofilm was improved for pulsed treatments (6 or 10 s) in the case of *L. plantarum* and without pulse for *B. longum* subsp. *infantis*; concerning power, an intensity at 50% (or at 30% for *L. plantarum*) improved biofilm formation and/or stability.

The effect was strongly strain dependent, as it was found only on *B. longum* subsp. *infantis* and on a strain of *L. plantarum*. Moreover, the results also suggest two possible effects: improvement of adhesion for *Bifidobacteria* and positive modulation of biofilm stability for *L. plantarum*.

These results are promising, although they should be confirmed by other assays, such as biofilm formation and adhesion to intestinal model cell lines or to other abiotic surfaces. However, since a strain-dependence was found, further investigations are required to understand the mechanisms behind the recovered ultrasonic modulation of microbial cell properties.

## Data availability statement

The raw data supporting the conclusions of this article will be made available by the authors, without undue reservation.

## Author contributions

MS, MC, and AB designed the study. AR, BS, CA, and AB performed the experiments. AR and AB wrote the manuscript and analyzed the data. MS, MC, CA, and BS reviewed and edited the manuscript. MS and MC funded the research. AB supervised the experiments. All authors contributed to the article and approved the submitted version.
